# Weighted gene co-expression network analysis to identify key modules and hub genes related to hyperlipidaemia

**DOI:** 10.1186/s12986-021-00555-2

**Published:** 2021-03-04

**Authors:** Fu-Jun Liao, Peng-Fei Zheng, Yao-Zong Guan, Hong-Wei Pan, Wei Li

**Affiliations:** 1 Department of Cardiology, Affiliated Hospital of Guizhou Medical University, 28 Guiyi Street, Guiyang, 550002 Guizhou People’s Republic of China; 2 Department of Cardiology, The Central Hospital of ShaoYang, 36 QianYuan lane, Shaoyang, 422000 Hunan People’s Republic of China; 3 Graduate School of Guangxi Medical University, 22 Shuangyong Road, Nanning, 530021 Guangxi People’s Republic of China; 4 Department of Cardiology, Hunan Provincial People’s Hospital and First Affiliated Hospital of Hunan Normal University, Changsha, Hunan People’s Republic of China

**Keywords:** Weighted gene co-expression network analysis, Hyperlipidaemia, Significant modules, Hub genes

## Abstract

**Background:**

The purpose of this study was to explore the potential molecular targets of hyperlipidaemia and the related molecular mechanisms.

**Methods:**

The microarray dataset of GSE66676 obtained from patients with hyperlipidaemia was downloaded. Weighted gene co-expression network (WGCNA) analysis was used to analyse the gene expression profile, and the royal blue module was considered to have the highest correlation. Gene Ontology (GO) functional and Kyoto Encyclopedia of Genes and Genomes (KEGG) pathway enrichment analyses were implemented for the identification of genes in the royal blue module using the Database for Annotation, Visualization and Integrated Discovery (DAVID) online tool (version 6.8; http://david.abcc.ncifcrf.gov). A protein–protein interaction (PPI) network was established by using the online STRING tool. Then, several hub genes were identified by the MCODE and cytoHubba plug-ins in Cytoscape software.

**Results:**

The significant module (royal blue) identified was associated with TC, TG and non-HDL-C. GO and KEGG enrichment analyses revealed that the genes in the royal blue module were associated with carbon metabolism, steroid biosynthesis, fatty acid metabolism and biosynthesis pathways of unsaturated fatty acids. *SQLE* (degree = 17) was revealed as a key molecule associated with hypercholesterolaemia (HCH), and *SCD* was revealed as a key molecule associated with hypertriglyceridaemia (HTG). RT-qPCR analysis also confirmed the above results based on our HCH/HTG samples.

**Conclusions:**

*SQLE* and *SCD* are related to hyperlipidaemia, and *SQLE/SCD* may be new targets for cholesterol-lowering or triglyceride-lowering therapy, respectively.

**Supplementary Information:**

The online version contains supplementary material available at 10.1186/s12986-021-00555-2.

## Background

With the development of markedly improved living standards throughout society, coronary artery disease (CAD) has emerged as a leading factor of mortality, functional deterioration, skyrocketing healthcare expenditure, disability and morbidity. CAD contributes to roughly 30% of all the deaths globally. It is estimated that its incidence will continue to rise in the coming decades [1–3]. Prior research has proved that the occurrence of CAD was the result of numerous factors comprising of genetic background, blood lipid levels, lifestyle, environmental exposures as well as the interactions between these factors [4, 5]. Coronary atherosclerosis is usually considered to be the initial step of CAD [6], which is due to the dysregulation of lipid metabolism and abnormal accumulation of cholesterol in the subintima layer of the coronary arteries [7, 8]. Hyperlipidaemia (HLP) acts as a critical risk factor that gives rise to CAD and its complications. Several investigations have demonstrated that for every 2% decrease in high-density lipoprotein cholesterol (HDL-C) levels, there is a resultant increase in CAD risk by 1%. Similarly, every 1% decrease in low-density lipoprotein cholesterol (LDL-C) levels results in reducing CAD risk by 1% [9, 10]. Several compelling studies have also demonstrated that combined effect in reducing the triglyceride (TG) [11], LDL-C [12] and total cholesterol (TC) [11] levels yielded higher decreases in cardiovascular risk compared to reduction of LDL-C levels alone [13]. The “6 percent effect” of statins refers to the fact that doubling the dose of statins only decreases LDL-C levels by 6.4%, and PCSK9 inhibitors combined with statins are recommended for patients with acute coronary syndrome (ACS) with a high risk of cardiovascular events [14]. Thus, the identification of novel therapeutic targets for HLP is expected to further reduce the risk of cardiovascular disease.

Microarray analysis might serve as a novel and practical approach to identify susceptibility genes associated with HLP [15]. However, the reproducibility and sensitivity of microarray analysis based on differentially expressed genes may be limited [16, 17]. Gene co-expression network-based methods have been widely used in processing microarray data and have especially been used to identify meaningful functional modules [18, 19]. Weighted gene co-expression network analysis (WGCNA) is one of the most effective methods of gene co-expression network analysis. Instead of simply identifying the differentially expressed genes, a scale-free network of gene–gene interactions is generated by WGCNA, and several significant modules composed of genes with similar functions could be identified by WGCNA; in addition, it can be used to further analyse the correlation between modules and phenotypes or clinical characteristics [20]. Therefore, WGCNA could be utilized to construct a co-expression network and identify significant modules in the network, which may help us to illuminate the intrinsic characteristics of HLP and provide new insights into potential genetic biomarkers, signalling pathways and molecular mechanisms involved in HLP.

## Materials and methods

### Hyperlipidaemia microarray datasets

The microarray dataset obtained from patients with HLP (GSE66676) was downloaded from the National Center for Biotechnology Information (NCBI) Gene Expression Omnibus (GEO, http://www.ncbi.nlm.nih.gov/geo/) database, which is based on the platform of the GPL6244 Affymetrix Human Gene 1.0 ST Array. Gene expression value matrices were obtained from the original files in CEL format after normalizing the expression values by using RMA methods in R software (version 4.0.0). [21]. Then, the Bioconductor package was used to transform the probe identification numbers (IDs) into gene symbols [22]. When multiple probe IDs corresponded to the same gene, the average expression value was used as the expression value.

### Construction of the weighted gene co-expression network

WGCNA is a widely used systems biology method that is usually used to establish a scale-free network based on gene expression data profiles [18]. The co-expression network was constructed by selecting the genes whose variance was greater than all the quartiles of variance. After the sample cluster tree was constructed, cut height = 35 was used to screen the samples for subsequent studies. To ensure the reliability of the results of the network construction, the outlier samples were eliminated, and the samples in cluster 1 were selected to build the sample dendrogram and trait heatmap. The appropriate soft threshold power (soft power = 9) was selected according to the standard scale-free networks, and the adjacency values between all differentially expressed genes were calculated using a power function. Then, the adjacency values were transformed into a topological overlap matrix (TOM), and the corresponding dissimilarity (1-TOM) values were calculated. Module identification was accomplished with the dynamic tree cut method by hierarchically clustering genes using 1-TOM as the distance measure with a minimum size cut-off of 30 and a deep split value of 2 for the resulting dendrogram. To verify the stability of the identified modules, a module preservation function was used to calculate module preservation and quality statistics in the WGCNA package [23].

### Identification of the module of interest and functional annotation

Pearson correlation analysis was used to assess the correlations between modules and clinical characteristics to identify biologically meaningful modules. All genes associated with the significant module were subjected to Gene Ontology (GO) and Kyoto Encyclopedia of Genes and Genomes (KEGG) pathway analyses by using the Database for Annotation, Visualization and Integrated Discovery (DAVID) online tool (version 6.8; http://david.abcc.ncifcrf.gov). *P* < 0.05 was set as the cut-off criterion.

### Hub gene analysis

The degree of module membership (MM) was defined as the correlation between the gene expression profile and the module eigengenes (Mes). The degree of gene significance (GS) was defined as the absolute value of the correlation between the gene and external traits. In general, modules with increased MS and GS values among all the identified modules were selected for further analysis of their biological function [24]. The protein–protein interaction (PPI) network of genes in the selected module was constructed by the Search Tool for the Retrieval of Interacting Genes database (version 11.0; http://www.string-db.org) [25] and then visualized using Cytoscape software [26]. Molecular complex detection (MCODE) [27] was used to identify the most valuable clustering module. An MCODE score > 4 was the threshold for inclusion in further analysis. CytoHubba, a Cytoscape plugin, was used to identify hub genes in the PPI network; it provides 11 methods to explore important nodes in biological networks, of which degree has a better performance [28].

### Sample verification and diagnostic criteria

A total of 462 (229 males, 49.57%; 233 females, 50.43%) unrelated participants with normal lipid levels and 485 (236 males, 48.66%; 249 females, 51.34%) unrelated subjects with hypercholesterolaemia (HCH, TC > 5.17 mmol/l) and 474 (232 males, 49.16%; 241 females, 50.84%) unrelated participants with hypertriglyceridaemia (HTG, TG > 1.70 mmol/l) were randomly recruited from the Physical Examination Center of the Affiliated Hospital of Guizhou Medical University. The age ranged from 24 to 82 years. There was no difference in age distribution or sex ratio between the control and HCH or HTG groups. Patients suffering from HCH did not have a history of HTG, and patients suffering from HTG did not have a history of HCH. All participants were basically healthy and had no history of myocardial infarction, CAD, type 2 diabetes mellitus (T2DM) or ischaemic stroke. They were not taking any medicines that could alter serum lipid levels. All subjects had signed written informed consent. The research protocol was approved by the Ethics Committee of the Affiliated Hospital of Guizhou Medical University.

### Epidemiological analysis

Universally standardized methods and protocols were used to conduct the epidemiological survey [29]. Detailed lifestyle and demographic characteristics were collected with a standard set of questionnaires. Alcohol consumption (0 (non-drinker), < 25 g/day and ≥ 25 g/day) and smoking status (0 (non-smoker), < 20 cigarettes/day and ≥ 20 cigarettes/day) were divided into three different subgroups. Waist circumference, BMI, height, blood pressure and weight were measured as previously described [30].

### Biochemical assays

Fasting venous blood samples of 5 ml were collected from each subject. A portion of the sample (2 ml) was placed in a tube and used to measure serum lipid levels. The remaining sample (3 ml) was collected in a glass tube containing anticoagulants (14.70 g/L glucose, 13.20 g/L trisodium citrate, 4.80 g/L citric acid) and utilized to extract deoxyribonucleic acid (DNA). The methods for performing serum ApoA1, HDL-C, ApoB, TG, LDL-C and TC measurements were described in a previous study [31]. All determinations were conducted using an autoanalyser (Type 7170A; Hitachi Ltd., Tokyo, Japan) in the Clinical Science Experiment Center of the Affiliated Hospital of Guizhou Medical University.

### Quantitative real-time PCR

Peripheral blood monocytes (PBMCs) were isolated from blood samples with TRIzol reagent, which was used to extract the total RNA that was then reverse-transcribed into cDNA by using the PrimeScript RT reagent kit (Takara Bio, Japan). The obtained cDNA was used as a template for RT-qPCR. Table 1 shows that specific primer sequences, which were designed by Sangon Biotech (Shanghai, China), were used to detect the 2 hub genes. Quantitative RT-PCR was performed using a Taq PCR Master Mix Kit (Takara) on an ABI Prism 7500 sequence-detection system (Applied Biosystems, USA) using RT Reaction Mix in a total volume of 20 μL with the following reaction conditions: pre-denaturation at 95 °C for 30 s, then 40 cycles of 95 °C for 30 s and 60 °C for 30 s.

**Table 1:** PCR primers for quantitative real-time PCR

Gene	Forward primer	Reverse primer
SQLE	TCTGGGGGTTAAGAGCAGTG	GTGTCTACACTTACCATCTGTGGC
SCD	CTTGCGATATGCTGTGGTGC	GGCTCCTAGCCTAATCCCCT
GAPDH	GCAACTAGGATGGTGTGGCT	TCCCATTCCCCAGCTCTCATA

### Diagnostic criteria

The values of serum ApoB (0.80–1.05 g/L), HDL-C (1.16–1.42 mmol/L), ApoA1 (1.20–1.60 g/L), TC (3.10–5.17 mmol/L), TG (0.56–1.70 mmol/L), the ApoA1/ApoB ratio (1.00–2.50) and LDL-C (2.70–3.10 mmol/L) were defined as normal at our Clinical Science Experiment Center. Subjects with TG > 1.70 mmol/L were defined as having hypertriglyceridaemia, and TC > 5.17 mmol/L was defined as having hypercholesterolaemia [32]. Participants with a fasting plasma (blood) glucose value ≥ 7.0 mmol/L were defined as having diabetes [33]. The diagnostic criteria of hypertension [34], obesity, normal weight and overweight were described in our previous study [35].

### Statistical analyses

SPSS (Version 22.0) was used to process the research data. The results are presented as the mean ± SD except for TG levels, which are presented as medians and interquartile ranges. The differences in the general characteristics except for TG between HCH/HTG patients and controls were analysed by independent-samples t tests. The Kruskal–Wallis and Mann–Whitney nonparametric tests were used to detect the difference in TG levels between patients with HCH/HTG and controls. The chi-square test was utilized to assess the differences in the proportion of smokers, age distribution and alcohol consumption between patients with HCH/HTG and controls. Heat mapping of the correlation models and bioinformatic analysis were performed in R software (version 4.0.0). A *P* value < 0.05 was considered to be statistically significant.

## Results

### Data pre-processing

Gene expression profiles were obtained after normalization of the data and removing the outliers, and a total of 20,284 gene symbols were identified from 67 samples. Additional details about the gene expression profile and the sample phenotypes are presented in Additional file 1: Tables S1 and S2.

### Weighted gene co‑expression networks

**Fig. 1:**
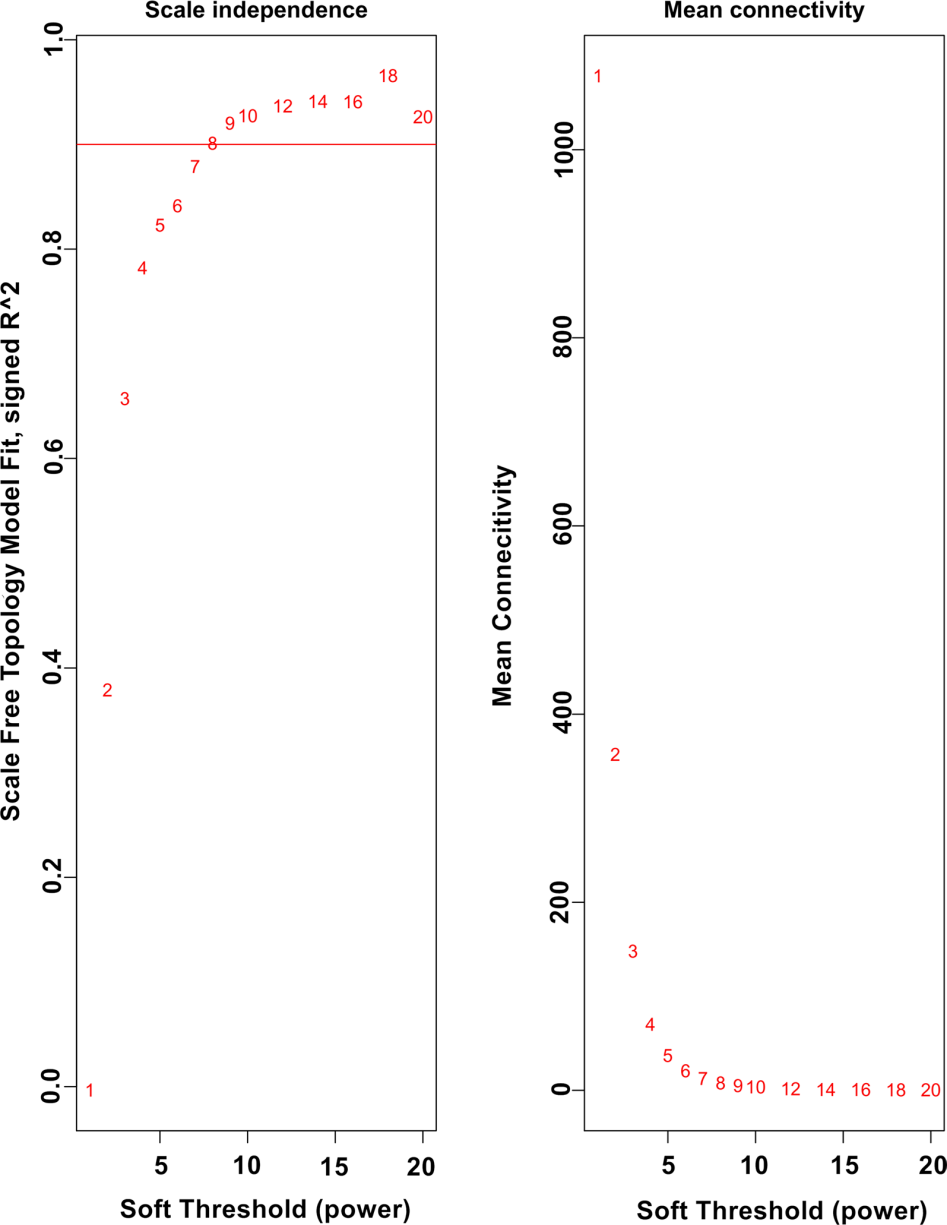
Analysis of network topology for various soft-thresholding powers. The left panel shows the scale-free fit index (y-axis) as a function of the soft-thresholding power (x-axis). The right panel displays the mean connectivity (degree, y-axis) as a function of the soft-thresholding power (x-axis)

**Fig. 2:**
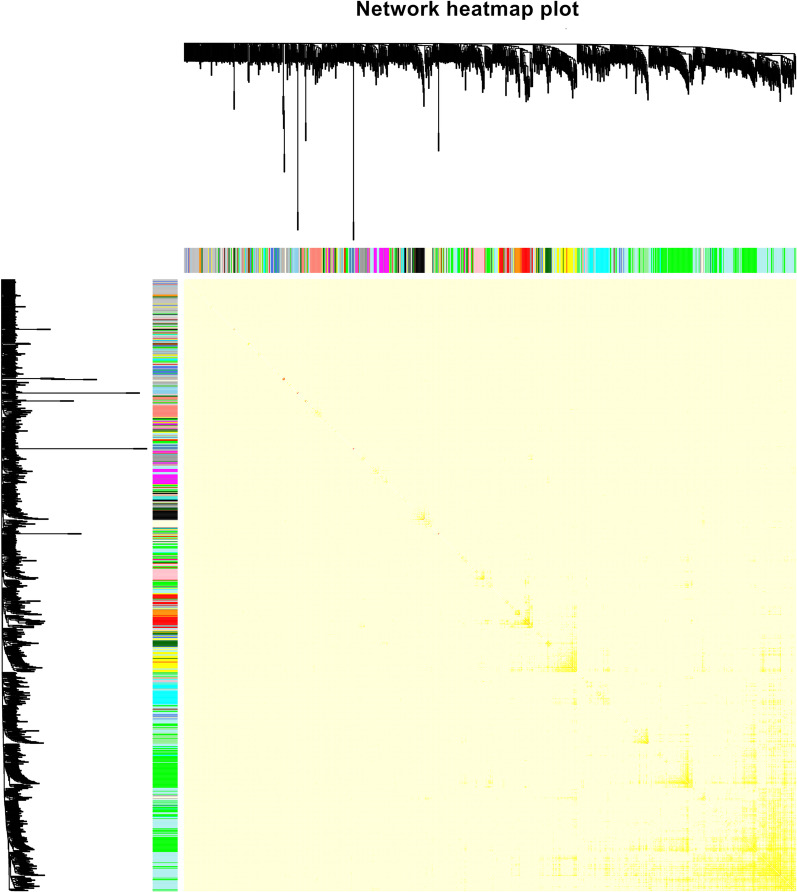
Heatmap plot of the topological overlap in the gene network. In the heatmap, each row and column correspond to a gene, light colour denotes low topological overlap, and progressively darker red denotes higher topological overlap. Darker squares along the diagonal correspond to modules. The gene dendrogram and module assignment are shown along the left and top

**Fig. 3:**
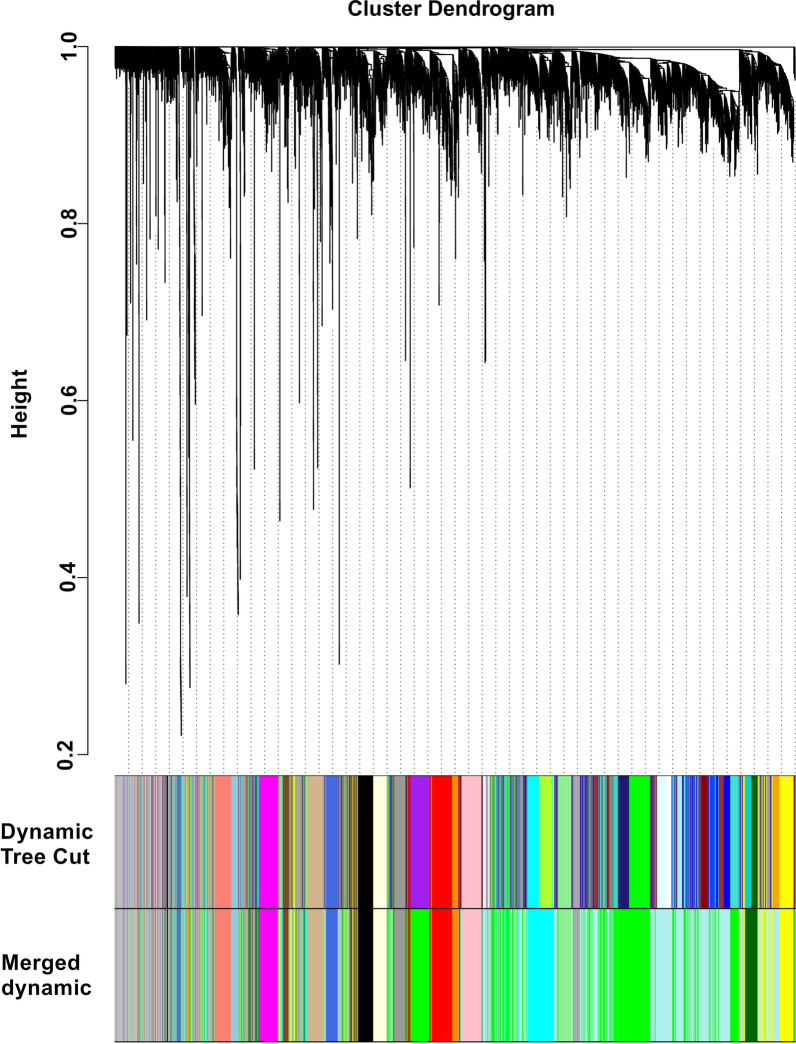
Clustering dendrogram of genes. Gene clustering tree (dendrogram) obtained by hierarchical clustering of adjacency-based dissimilarity. The coloured row below the dendrogram indicates module membership identified by the dynamic tree cut method, together with assigned merged module colours and the original module colours

The sample cluster tree and sample dendrogram and trait heatmap are shown in Additional file 2: Figures S1 and S2. The gene expression profiles of 42 samples in cluster 1 were selected to build the weighted gene co-expression network. After the soft threshold (β = 9) was determined (Fig. 1), the weighted gene co-expression network was constructed by selecting the genes whose variance was greater than all the quartiles of variance. The adjacency matrix and correlation matrix of the gene expression profile were calculated and then transformed into a topological overlap matrix (TOM), and a clustering tree of genes based on the gene–gene non-ω similarity was obtained (Fig. 2). Combined with the TOM, the gene modules of each gene network were identified by the hierarchical average linkage clustering method, and twenty gene modules were identified by the dynamic tree cut algorithm (cut height = 0.25) (Fig. 3). The grey module contains all the genes that do not belong to the other modules and were excluded from subsequent analysis.

### Identification of the modules of interest and functional annotation

**Fig. 4:**
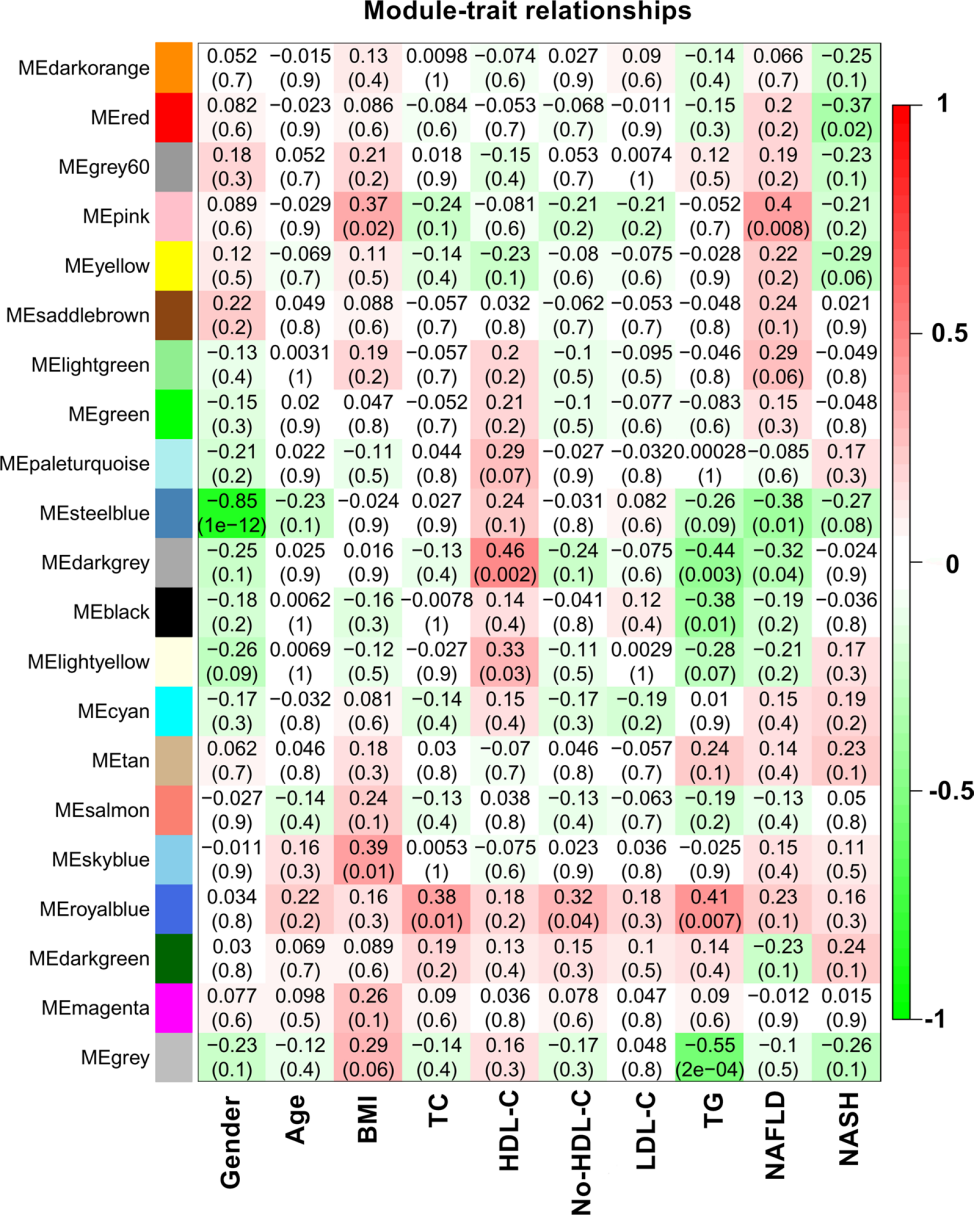
Module-feature associations. Each row corresponds to a module Eigengene, and the column corresponds to the clinical phenotype. Each cell contains the corresponding correlation in the first line and the P-value in the second line. The table is colour-coded by correlation according to the colour legend

**Fig. 5:**
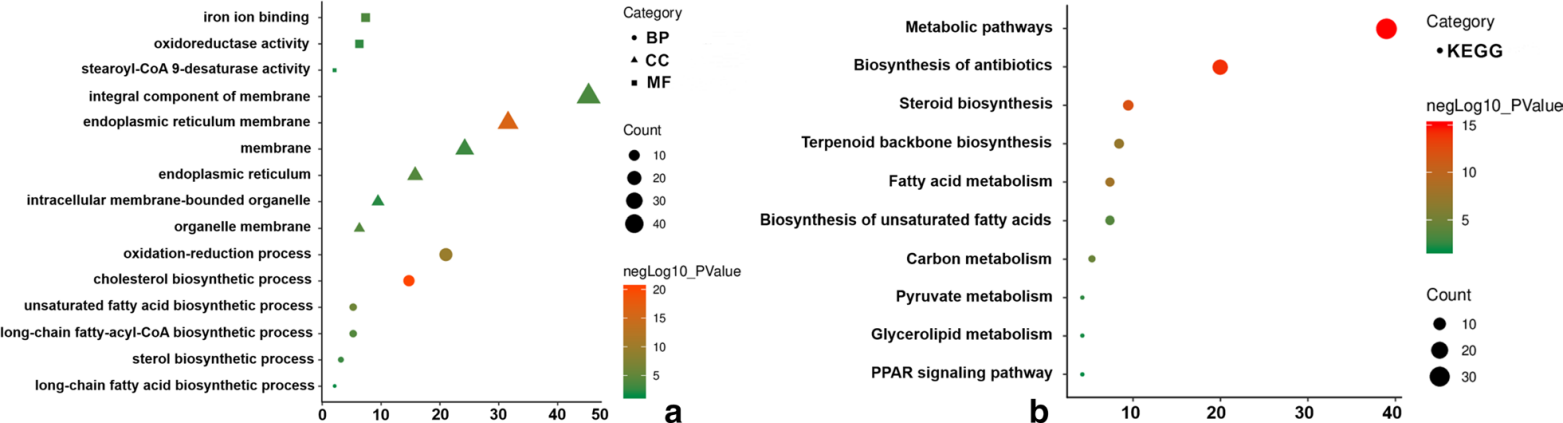
GO functional and KEGG pathway enrichment analyses for genes in the object module. The x-axis shows the number of genes, and the y-axis shows the GO and KEGG pathway terms. The -log10 (P-value) of each term is coloured according to the legend. (**A**): GO functional enrichment analysis. (**B**): KEGG pathway enrichment analysis

The identification of modules that were significantly related to clinical phenotype was of high biological significance. In this study, we noticed that the royal blue module was associated with *TC* (*r *^*2*^ = 0.38, *P* = 0.01), TG (*r *^*2*^ = 0.41, *P* = 0.007) and non-HDL-C (*r *^*2*^ = 0.32, *P* = 0.04), and the genes in the royal blue module were studied in the subsequent analyses (Fig. 4). GO and KEGG pathway enrichment analyses were used to further explore the biological functions of the genes in the royal blue module. Furthermore, we noticed that a total of 101 genes (Additional file 1: Tables S3) in the royal blue module were significantly correlated with the following pathways: hsa01100: metabolic pathways, hsa01130: biosynthesis of antibiotics, hsa00100: steroid biosynthesis, hsa01212: fatty acid metabolism, and hsa01040: biosynthesis of unsaturated fatty acids. The cell components, biological processes, molecular functions and KEGG pathway analysis of the royal blue module are also shown in Fig. 5, and more detailed information is presented in Additional file 1: Tables S4 and S5.

### PPI network construction and module analysis of DEGs

**Fig. 6:**
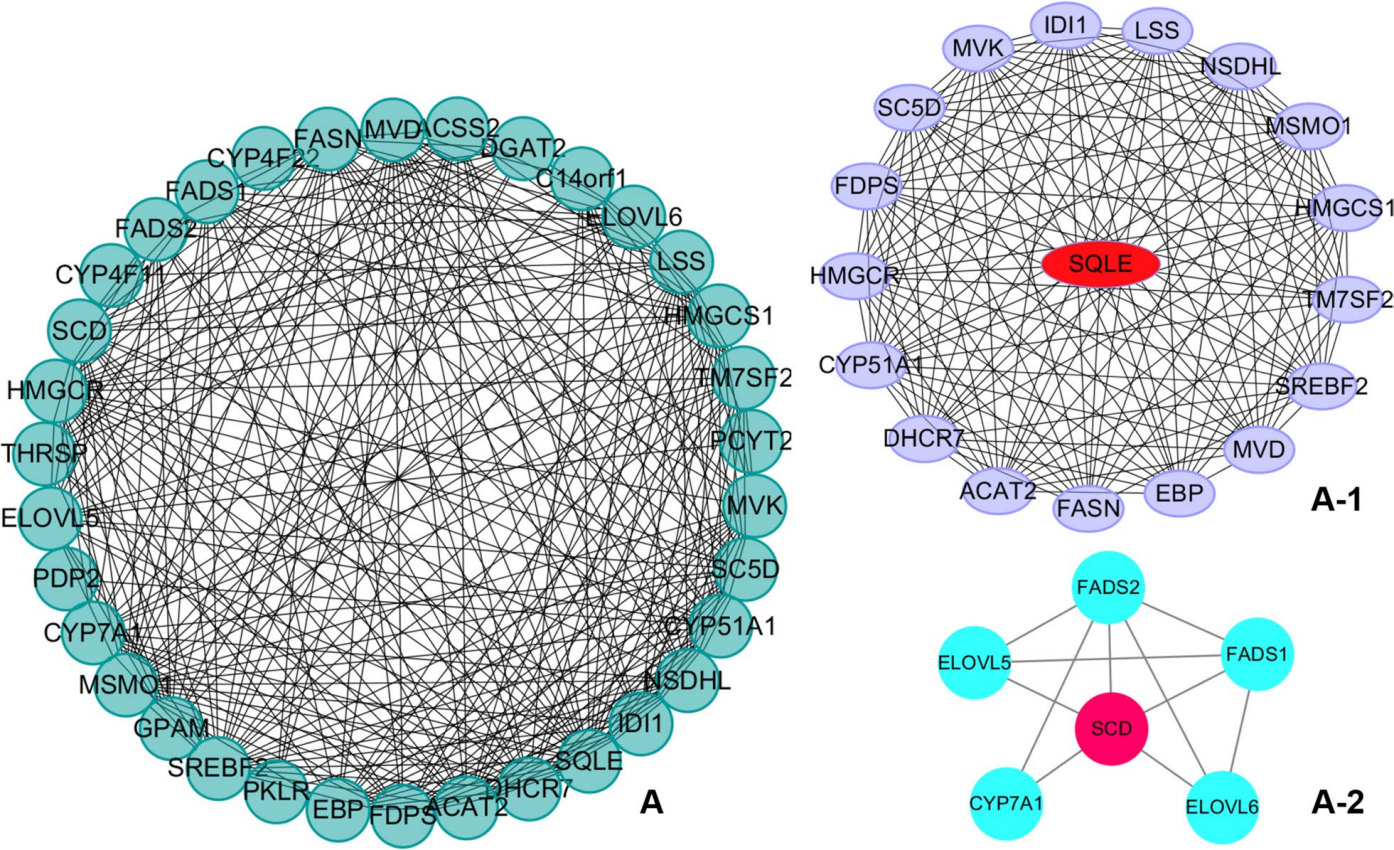
PPI network construction and identification of hub genes. (**A**) PPI network of genes in the royal blue module. The edge shows the interaction between two genes. Significant modules identified from the PPI network using MCODE with a score > 4.0. (**A-1**) Molecular-1 with MCODE score = 17.29. (**A-2**) Molecular-2 with MCODE score = 4.4

A PPI network including 93 notes and 333 edges was constructed by the STRING online tool. As shown in Fig. 6, the hub genes *SQLE* (degree = 17) and *SCD* (degree = 5) were identified by cytoHubba plug-ins in Molecular-1 and Molecular-2, respectively. Thus, we speculate that the genes mentioned above may be significantly correlated with blood lipid metabolism.

### Validation analysis by RT-qPCR

**Fig. 7:**
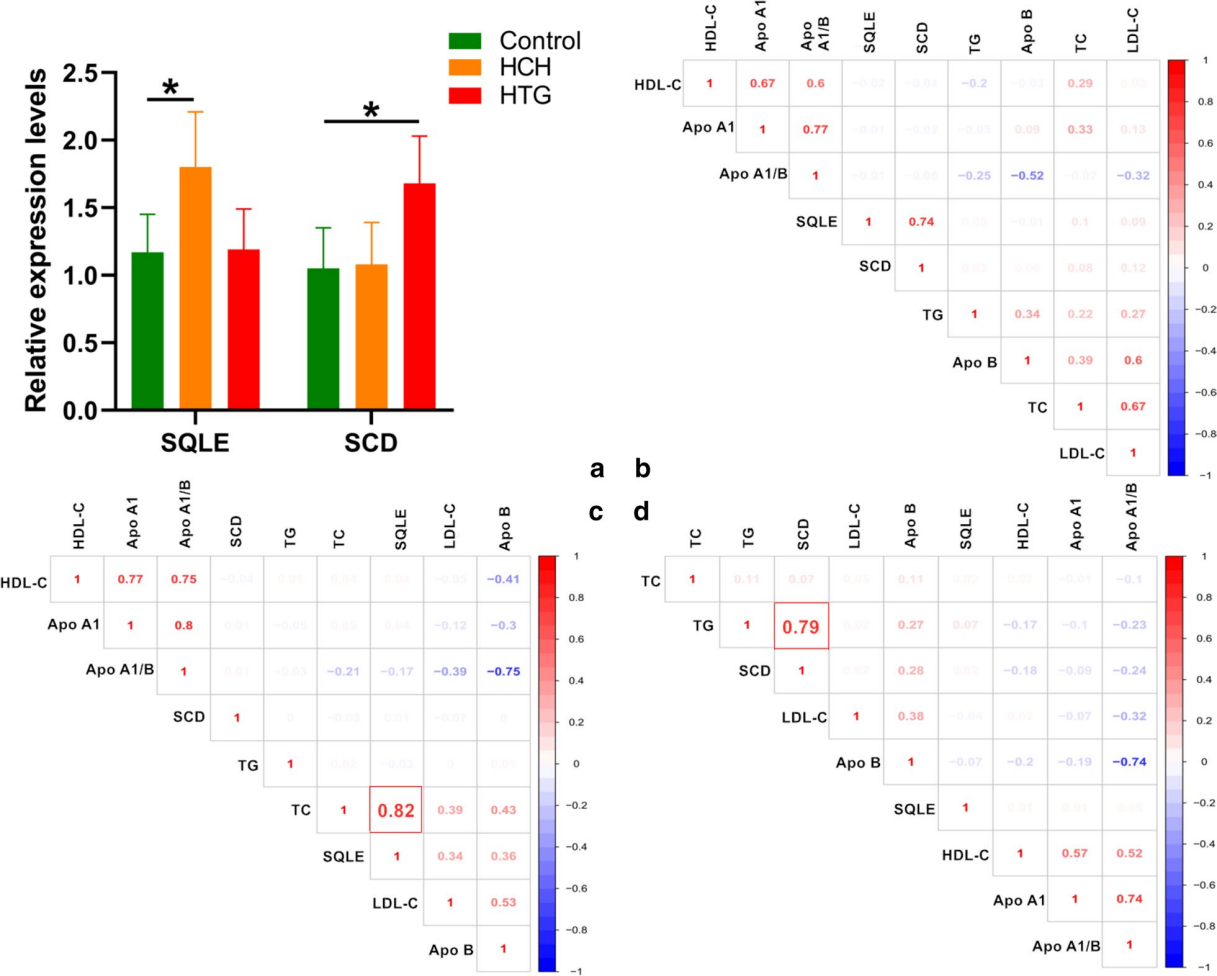
Validation with RT-qPCR (**a**) and the relationship between genes and lipid parameters in the control (**b**), HCH (**c**) and HTG (**d**) groups. *P < 0.001

As shown in Fig. 7a, the RT-qPCR results revealed that the expression of *SQLE* in the HCH group and *SCD* in the HTG group was higher than that in healthy subjects. At the same time, we also noticed that *SQLE* was positively correlated with TC (Fig. 7c) levels in the HCH group and that *SCD* was positively correlated with TG levels in the HTG group (Fig. 7d).

### Common and biochemical characteristics

As mentioned in Table 2, the sex ratio, age and height were similar between the controls and patients with HCH/HTG. Serum HDL-C and ApoA1 levels and the ApoA1/ApoB ratio were significantly higher, and the proportion of smokers, proportion of drinkers, systolic blood pressure, waist circumference, weight, diastolic blood pressure, glucose level, pulse pressure, body mass index (BMI), and serum LDL-C, ApoB, TG and TC levels were significantly lower in controls than in patients with hyperlipidaemia.

**Table 2:** Comparison of demographic, lifestyle characteristics and serum lipid levels of the participants

Characteristic	Control (n = 462)	HCH (n = 485)	HTG (n = 474)	*P*_*HCH vs. controls*_	*P*_*HTG vs. controls*_
Male/female^3^	229/233	236/249	232/241	0.780	0.870
Age (years)^1^	57.60 ± 8.81	58.13 ± 9.69	57.10 ± 7.61	0.379	0.359
Height (cm)^1^	159.83 ± 8.20	160.66 ± 7.93	159.92 ± 8.11	0.114	0.771
Weight (kg)^1^	58.98 ± 9.90	62.66 ± 10.09	60.52 ± 11.08	1.97E−8	0.021
Body mass index (kg/m^2^)^1^	23.05 ± 3.32	24.21 ± 3.12	23.61 ± 3.67	3.59E−8	0.014
Waist circumference^1^	74.50 ± 8.47	78.37 ± 8.76	81.10 ± 9.21	1.00E−11	4.10E−28
Smoking, *n* %^3^					
Non-smoker	355	331	349		
≤ 20 cigarettes/day	98	114	73		
> 20 cigarettes/day	9	40	52	1.73E−5	4.42E−8
Alcohol, *n* %^3^					
Non-drinker	377	354	363		
≤ 25 g/day	45	55	44		
> 25 g/day	40	76	67	0.002	0.031
SBP (mmHg)^1^	135.49 ± 22.58	139.52 ± 22.56	141.15 ± 20.42	0.006	1.08E−4
DBP (mmHg)^1^	82.45 ± 12.42	84.01 ± 11.71	85.15 ± 11.72	0.047	0.001
PP (mmHg)^1^	53.04 ± 17.77	55.51 ± 18.19	56.00 ± 14.43	0.035	0.007
Glu (mmol/L)^1^	6.14 ± 1.42	6.44 ± 1.58	6.35 ± 1.32	0.002	0.015
TC (mmol/L)^1^	4.37 ± 0.64	5.80 ± 0.50	4.46 ± 0.37	9.42E−47	0.007
TG (mmol/L)^2^	0.99(0.53)	1.15(0.45)	2.30(1.03)	4.46E−9	1.69E−84
HDL-C (mmol/L)^1^	1.64 ± 0.48	1.48 ± 0.43	1.44 ± 0.46	2.56E−8	7.22E−11
LDL-C (mmol/L)^1^	2.50 ± 0.55	3.52 ± 0.80	2.79 ± 0.86	4.89E−32	1.86E−9
ApoA1 (g/L)^1^	1.33 ± 0.24	1.23 ± 0.25	1.25 ± 0.24	3.49E−11	4.66E−8
ApoB (g/L)^1^	0.98 ± 0.17	1.02 ± 0.18	1.07 ± 0.21	0.002	1.06E−11
ApoA1/ApoB^1^	1.39 ± 0.33	1.25 ± 0.40	1.22 ± 0.39	1.91E−8	7.62E−12

*SBP* Systolic blood pressure; *DBP* Diastolic blood pressure; *PP* Pulse pressure; *Glu* Glucose; *HDL-C* high-density lipoprotein cholesterol; *LDL-C* low-density lipoprotein cholesterol; *Apo* Apolipoprotein; *TC* Total cholesterol*; TG* Triglyceride

^1^Mean ± SD determined by t-test

^2^Median (interquartile range) tested by the Wilcoxon–Mann–Whitney test

^3^The rate or constituent ratio between the different groups was analyzed by the chi-square test

## Discussion

Several recent studies have shown that hypertension, smoking, obesity, age, dyslipidaemia, lack of exercise, sex and diabetes mellitus are common risk factors for cardiovascular disease [36, 37]. A comprehensive understanding of the potential molecular mechanisms involved in the pathogenesis of HLP is helpful for its prevention and treatment. As a novel and practical approach to the identification of HLP susceptibility genes, a microarray analysis using WGCNA may be helpful for the diagnosis of hyperlipidaemia [20]. WGCNA could be used to build a scale-free co-expression network of lipid-associated genes by detecting gene-to-gene interactions rather than simply focusing on the differentially expressed genes (DEGs). Co-expressed genes were enriched in different modules by hierarchical average linkage cluster analysis. In the present research, we analysed a dataset from HLP patients (GSE66676) by using WGCNA and identified that the royal blue module was significantly associated with TC, TG and non-HDL. Furthermore, KEGG enrichment analyses of the genes in the royal blue module indicated that the enriched genes in this module might have significant potential biological functions that are closely related to metabolic pathways, steroid biosynthesis, fatty acid metabolism and biosynthesis of unsaturated fatty acids. Two hub genes (*SQLE* and *SCD*) were identified in the royal blue module that were detected by MCODE analysis. Moreover, the verification results were highly consistent with the above findings, and we found that the expression of the *SQLE* gene in patients with HCH and the *SCD* gene in patients with HTG was higher than that in healthy controls. Therefore, the identified *SQLE* gene was associated with the onset of HCH, the *SCD* gene was associated with the onset of HTG, and the underlying molecular mechanisms of these genes might be slightly different. In addition, *SQLE* and *SCD* were previously reported to be statin responsive, and they are known to be involved in sterol metabolism and transport; at the same time, there were significant changes in expression levels in the B-cells in response to statin treatment [38], and therefore, *SQLE* and *SCD* may be new targets for lipid-lowering therapy.

Fatty acids and cholesterol are essential lipids involved in many crucial biological processes; however, excessive free fatty acids and free cholesterol are major risk factors for type 2 diabetes and atherosclerosis [39]. Previous studies on intermediate metabolites in cholesterol biosynthesis have shown that the first oxidative step in cholesterol biosynthesis is catalysed by squalene monooxygenase (*SQLE*), a crucial regulator downstream of HMG-CoA reductase (*HMGCR*) in cholesterol synthesis [40]. Meanwhile, *SQLE* is suggested as the second rate-limiting enzyme in cholesterol synthesis [41, 42]. Inhibition of *SQLE* expression could effectively reduce cholesterol synthesis [43, 44], and the cholesterol-lowering effect is caused by the combination of multiple levels. First, *SQLE* and *HMGCR* act as direct targets of the sterol regulatory element binding protein 2 (*SREBP2)* transcription factor and play a crucial regulatory role in most cholesterol biosensor genes [45, 46]. Second, the N-terminus of the *SQLE* protein may contain a cholesterol-sensitive region that mediates the protease degradation of *SQLE* in a cholesterol-dependent manner by relying on an E3 ubiquitin ligase such as *MARCH* [47]. Interestingly, oleate acts as an unsaturated fatty acid and can stabilize *SQLE* by blocking *MARCH6*-mediated degradation [48]. In addition, Masanori Honsho et al. also noticed that inhibition of *SQLE* expression through elevating plasmalogen levels may be a novel and alternative potential method to reduce cholesterol synthesis [40]. Similarly, the KEGG analyses in the current study indicated that *SQLE* was mainly involved in metabolic pathways and steroid biosynthesis.

Metabolic risk factors such as insulin resistance, obesity, hypertension and dyslipidaemia are correlated with each other, so their combination is generally referred to as “metabolic syndrome” (MetS). Abnormal stearoyl-coenzyme A desaturase (*SCD*) expression/activity has been noticed in subjects with metabolic syndrome, indicating that *SCD* may be related to the pathogenesis of metabolic syndrome. By querying the GENE database in NCBI, we noticed that *SCD* (also known as *SCD1*; *FADS5*; *SCDOS*; *hSCD1*; *MSTP008*; gene ID: 6319, HGNC: 10571, OMIM: 604031) is positioned on chromosome 10q24.31 (exon count: 6) and encodes a biological synthase, which is mainly involved in the metabolism of fatty acids, especially oleic acid. This protein is an intact membrane protein located in the endoplasmic reticulum and is a member of the fatty acid desaturase family. Herman-Edelstein M et al. proved that *SREBPs* are transcription factors that activate the synthesis of fatty acids (FAs), triglycerides (TGs), and cholesterol, and *SREBP2* activates cholesterol production, whereas *SREBP1* primarily activates FA and TG synthesis [49]. ATP-citrate lyase (*ACLY*), a cytosolic enzyme that generates acetyl-CoA for cholesterol and de novo fatty acid synthesis, is a potential target for hyperlipidaemic intervention [50]. *ACLY* acts as a critical enzyme involved in de novo fatty acid synthesis and catalyses the conversion of citrate to cytosolic acetyl-CoA. Acetyl-CoA is converted to malonyl CoA via acetyl-CoA carboxylase (*ACC*), which plays a key role in the first committed step in the synthesis of fatty acids [51]. *SCD* is another key rate-limiting enzyme in fatty acid metabolism downstream of *ACLY*; it can convert different saturated fatty acids into monounsaturated fatty acids, and its expression is directly regulated by *SREBP1* [52–55]. Both animal and human studies have shown that *SCD* is associated with obesity and insulin resistance [56, 57]. Mice with the *SCD* gene exhibited reduced diet-induced weight gain and improved insulin resistance compared to wild-type controls [58]. Deletion of the *SCD1* gene product in mice could effectively improve insulin sensitivity, reduce plasma non-HDL cholesterol and triglyceride levels and liver lipid accumulation and increase beneficial HDL cholesterol levels [59]. Daniel Castellano-Castillo et al. also found a negative relationship between *SCD* DNA methylation and BMI and the MetS index [60]. In the current study, we also noticed that *SCD* was mainly involved in fatty acid metabolism and the biosynthesis pathways of unsaturated fatty acids.

Several recent studies have indicated that smoking [61, 62] and excessive drinking [63] were associated directly to HLP development and progression along with its complications. In recent years, the influence of smoking on HLP has attracted increasing attention. A compelling research has indicated the existence of lower HDL-C and higher TC, LDL-C and TG levels in smokers than in non-smokers [61]. In addition, atherosclerosis formation has also been shown to be influenced by different alcohol doses [64]. Moderate alcohol consumption may be protective against cardiovascular events, a phenomenon that has been attributed to elevated levels of ApoA1 and HDL-C [65]. Nevertheless, frequent heavy drinking leading to dyslipidaemia, alcoholic fatty liver and abnormal liver function is known to increase risk of CAD mortality [66]. In the present study, we found that the percentage of participants who smoking and excessive drinking was greater in the hyperlipidaemic group than in the normal group. Therefore, the preventive effect of a healthy lifestyle on hyperlipidaemia should not be ignored when exploring new therapeutic targets for hyperlipidaemia.

This research had several limitations. First, this is a single-centre study comprising a small patient number, and large multicentre studies are necessary to validate our findings. Second, the molecular mechanisms of *SQLE* and *SCD* involved in HLP are still not fully defined and require further cytology and animal experiments to further outline their respective roles in vivo and in vitro.

## Conclusions

WGCNA identified that the royal blue module was significantly associated with TC, TG and non-HDL. GO and KEGG enrichment analyses revealed that the hub genes of *SQLE* were associated with TC and that *SCD* was associated with TG metabolism. The verification results of RT-qPCR revealed that the expression of *SQLE* in hypercholesterolaemia and SCD in hypertriglyceridaemia was higher than that in normal controls, which further increased the credibility of the conclusion. Thus, we speculated that *SQLE* may be a novel target for cholesterol-lowering therapy and that *SCD* may be a novel target for triglyceride-lowering therapy.

## Supplementary Information

**Additional file 1.** More details in Gene expression profile (Additional Table S1), Clinical phenotype (Additional Table S2), Module Genes (Additional Table S3), GO functional enrichment (Additional Table S4) and KEGG pathway enrichment analyses (Additional Table S5).

**Additional file 2.** The Figures of Clustering dendrogram of samples, and Sample dendrogram and trait heatmap of selected samples in cluster 1.

## Data Availability

The datasets used and/or analysed during the current study are available from the corresponding author on reasonable request.

## Abbreviations

WGCNA: Weighted gene co-expression network analysis
HCH: Hypercholesterolemia
HTG: Hypertriglyceridemia
SQLE: Squalene epoxidase
SCD: Stearoyl-CoA desaturase
DAVID: Database for Annotation, Visualization and Integrated Discovery
T2DM: Type 2 diabetes mellitus
GO: Gene Ontology
HDL-C: High-density lipoprotein cholesterol
IS: Ischemic stroke
KEGG: Kyoto Encyclopedia of Genes and genomes
LDL-C: Low-density lipoprotein cholesterol
MCODE: Molecular Complex Detection
Apo: Apolipoprotein
PPI: Protein–protein interaction
GEO: Gene Expression Omnibus
BMI: Body mass index
TG: Triglyceride
RT-qPCR: Quantitative real time polymerase chain reaction
TC: Total cholesterol
ACC: Acetyl CoA carboxylase
ACLY: ATP—Citrate Lyase
FAs: Fatty acids
MetS: Metabolic syndrome
HMGCR: HMG-CoA reductase
SREBP2: Sterol regulatory element binding protein 2
CAD: Coronary artery disease
HLP: Hyperlipidaemia
ACC: American College of Cardiology
AHA: American Heart Association
ACS: Acute coronary syndrome
TOM: Topological overlap matrix
MM: Module membership
Mes: Module eigengenes
Mes: Module eigengenes
DNA: Deoxyribonucleic acid
PBMCs: Peripheral blood monocytes
DEGs: Differentially expressed genes
