# Supplementary material for: Weighted gene co-expression network analysis to identify key modules and hub genes related to hyperlipidaemia
Source: Nutr Metab (Lond). 2021 Mar 4;18:24. doi: 10.1186/s12986-021-00555-2 (PMC7934476; doi:10.1186/s12986-021-00555-2)
Supplement: Supplementary file 2 — Additional file 2. The Figures of Clustering dendrogram of samples, and Sample dendrogram and trait heatmap of selected samples in cluster 1. [file 12986_2021_555_MOESM2_ESM.docx]

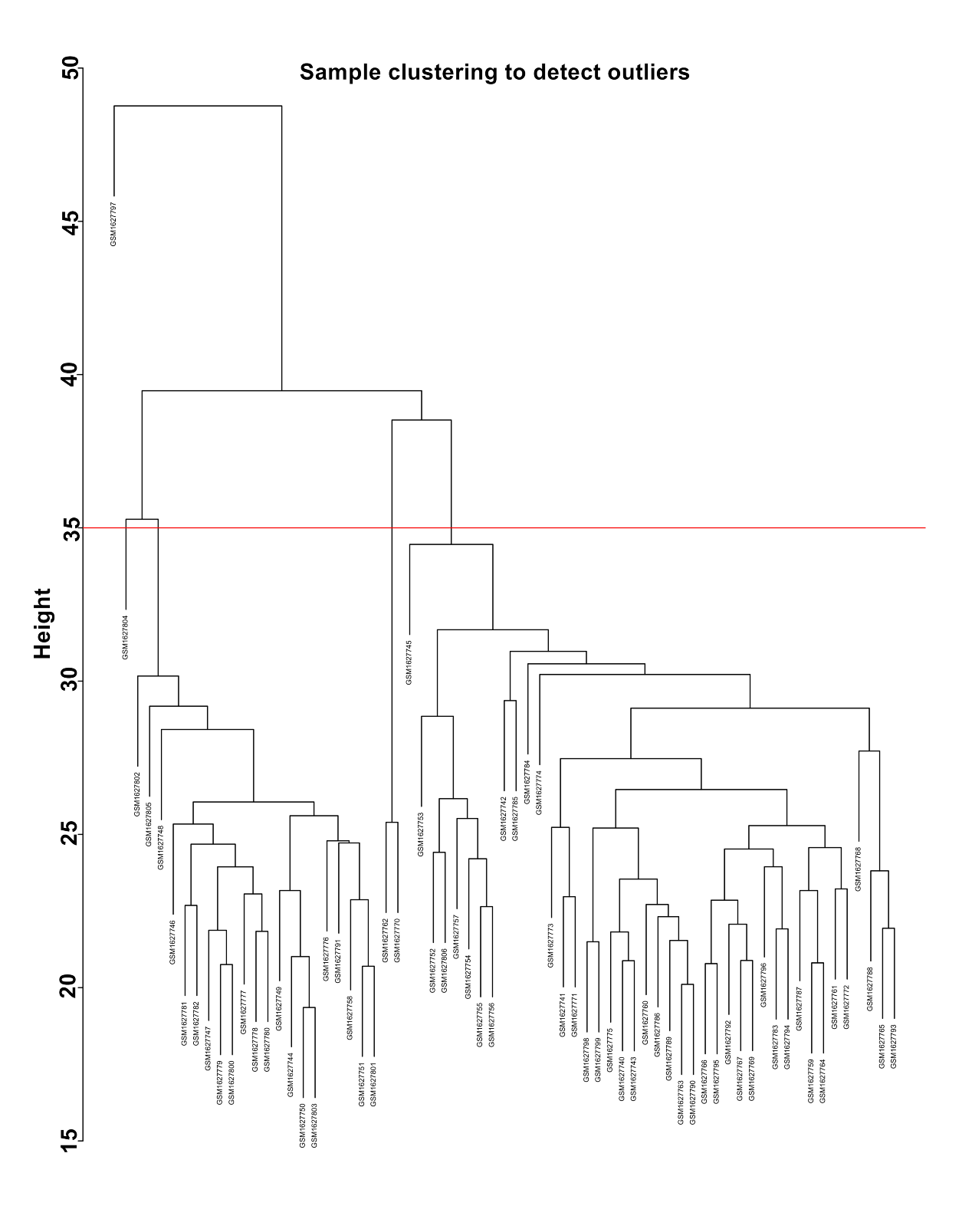


**Additional** **Figure S1: Clustering dendrogram of samples.**

Cut height = 35 was used to divide 67 samples into three different cluster types. Cluster 0 contains the following samples (GSM1627762, GSM1627770, GSM1627797, GSM1627804), cluster 1 contains the following samples (GSM1627740, GSM1627741, GSM1627742, GSM1627743, GSM1627745, GSM1627752, GSM1627753, GSM1627754, GSM1627755, GSM1627756, GSM1627757, GSM1627759, GSM1627760, GSM1627761, GSM1627763, GSM1627764, GSM1627765, GSM1627766, GSM1627767, GSM1627768, GSM1627769, GSM1627771, GSM1627772, GSM1627773, GSM1627774, GSM1627775, GSM1627783, GSM1627784, GSM1627785, GSM1627786, GSM1627787, GSM1627788, GSM1627789, GSM1627790, GSM1627792, GSM1627793, GSM1627794, GSM1627795, GSM1627796, GSM1627798, GSM1627799, GSM1627806) and cluster 2 contains the following samples (GSM1627744, GSM1627746, GSM1627747, GSM1627748, GSM1627749, GSM1627750, GSM1627751, GSM1627758, GSM1627776, GSM1627777, GSM1627778, GSM1627779, GSM1627780, GSM1627781, GSM1627782, GSM1627791, GSM1627800, GSM1627801, GSM1627802, GSM1627803, GSM1627805)


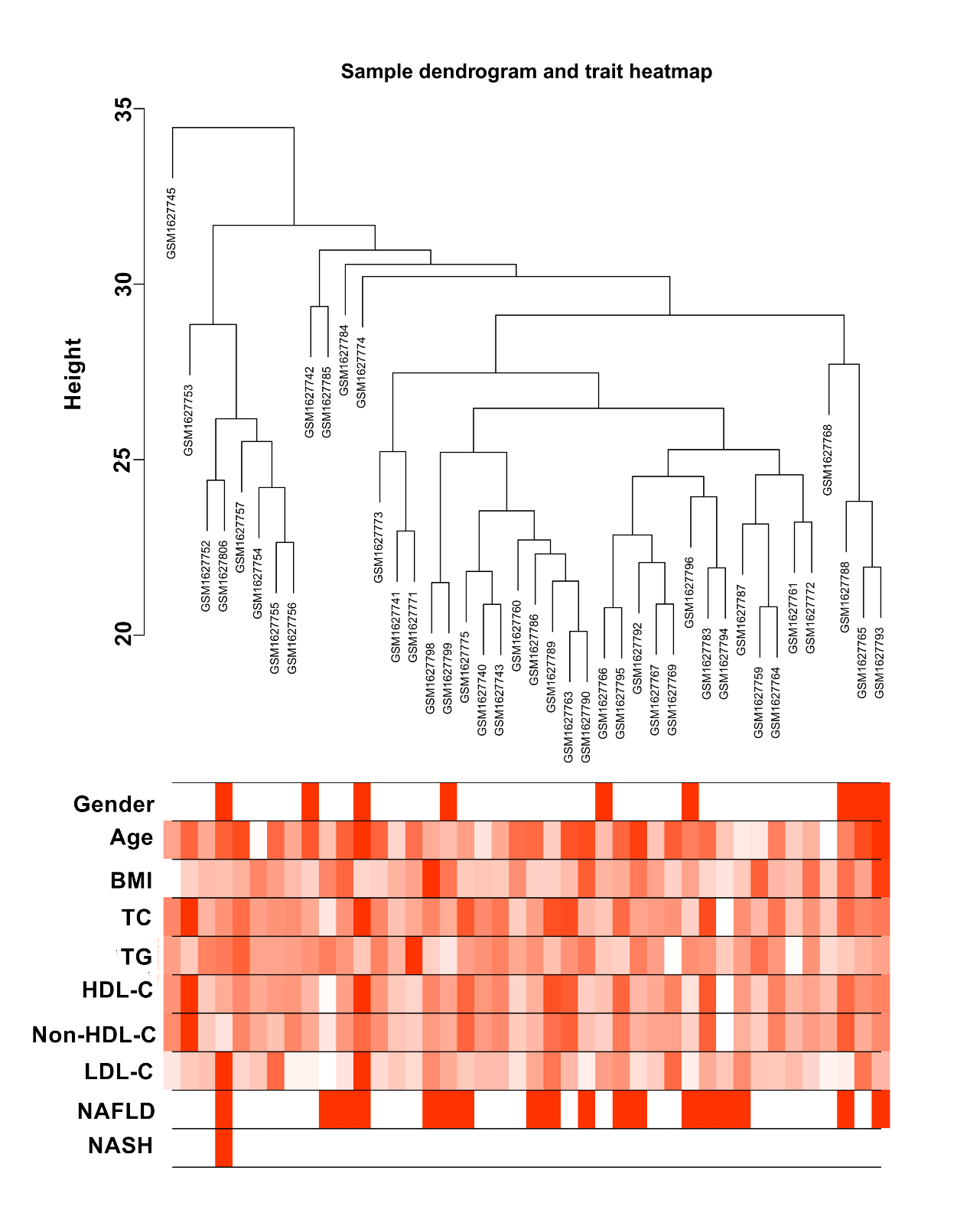


**Additional Figure S2: Sample dendrogram and trait heatmap of selected samples in cluster 1.**

*TC*, Total cholesterol; *TG*, Triglyceride; *HDL-C*, High-density lipoprotein cholesterol; *LDL-C*, Low-density lipoprotein cholesterol; *Non-HDL-C*, Non-High-density lipoprotein cholesterol
